# Gut Microbiome: Profound Implications for Diet and Disease

**DOI:** 10.3390/nu11071613

**Published:** 2019-07-16

**Authors:** Ronald D. Hills, Benjamin A. Pontefract, Hillary R. Mishcon, Cody A. Black, Steven C. Sutton, Cory R. Theberge

**Affiliations:** 1 Department of Pharmaceutical Sciences, College of Pharmacy, University of New England, Portland, ME 04103, USA; 2 Pharmacy Service, Boise Veterans Affairs Medical Center, Boise, ID 83702, USA; 3 College of Pharmacy, Ferris State University, Big Rapids, MI 49307, USA; 4 College of Pharmacy, University of Texas at Austin, San Antonio, TX 78229, USA

**Keywords:** gut microbiota, nutrition, habitual diets, Western diet, obesity, cardiometabolic risk factors, chronic health conditions, gastrointestinal disorders, prebiotics and probiotics

## Abstract

The gut microbiome plays an important role in human health and influences the development of chronic diseases ranging from metabolic disease to gastrointestinal disorders and colorectal cancer. Of increasing prevalence in Western societies, these conditions carry a high burden of care. Dietary patterns and environmental factors have a profound effect on shaping gut microbiota in real time. Diverse populations of intestinal bacteria mediate their beneficial effects through the fermentation of dietary fiber to produce short-chain fatty acids, endogenous signals with important roles in lipid homeostasis and reducing inflammation. Recent progress shows that an individual’s starting microbial profile is a key determinant in predicting their response to intervention with live probiotics. The gut microbiota is complex and challenging to characterize. Enterotypes have been proposed using metrics such as alpha species diversity, the ratio of Firmicutes to Bacteroidetes phyla, and the relative abundance of beneficial genera (e.g., *Bifidobacterium*, *Akkermansia*) versus facultative anaerobes (*E. coli*), pro-inflammatory *Ruminococcus*, or nonbacterial microbes. Microbiota composition and relative populations of bacterial species are linked to physiologic health along different axes. We review the role of diet quality, carbohydrate intake, fermentable FODMAPs, and prebiotic fiber in maintaining healthy gut flora. The implications are discussed for various conditions including obesity, diabetes, irritable bowel syndrome, inflammatory bowel disease, depression, and cardiovascular disease.

## 1. Introduction to Gut Microbiota and Disease

The intestinal microbiome has recently been implicated in a host of chronic diseases ranging from inflammatory bowel disease (IBD), type 2 diabetes (T2D), and cardiovascular disease (CVD) to colorectal cancer [1,2,3]. The community of ~200 prevalent bacteria, virus, and fungi inhabiting the human gastrointestinal (GI) tract provide unique metabolic functions to the host and are fundamentally important in health and disease [4,5]. Microbiome refers to the collective genomes of all microorganisms inhabiting an environment. While isolating and culturing each individual species is an intractable task, a cutting-edge method of sequence analysis, metagenomics, has enabled the reconstruction of microbial species and their function from the collective nucleotide contents contained in a stool sample. Shotgun metagenomic sequencing analysis discovered 1952 unclassified bacteria species in the human gut microbiome in addition to the 553 bacteria previously cultured from the gut [6]. A central question in medicine concerns the nature of the relationship between human health and the gut microbiota, which refers to the community of microorganisms themselves, the relative abundance of individual species populations, and their function.

Metagenomics and analysis of twins data has revealed that environmental factors such as diet and household cohabitation greatly outweigh heritable genetic contributions to the composition and function of gut microbiota [7]. Analogous to the genetic heritability statistic, Rothschild et al. constructed a microbiome-association index. Significant associations are observed between the gut microbiome and host phenotypes for body mass index (BMI) (25%), waist-to-hip ratio (24%), fasting glucose levels (22%), glycemic status (25%), high-density lipoprotein (HDL) cholesterol levels (36%), and monthly lactose consumption (36%) [7]. Compared to BMI, waist-to-hip ratio is an anthropometric measurement of central obesity and stronger predictor of diastolic and systolic blood pressure, total cholesterol/HDL, and triglycerides [8] as well as death from CVD [9].

The Western diet has profound effects on the diversity and populations of microbial species that make up gut flora [10]. The U.S. is home to the largest number of immigrants in the world, many of whom develop metabolic diseases post immigration. Earlier epidemiological evidence revealed a fourfold increase in obesity risk is possible within 15 years of emigrating to the U.S. compared to populations remaining in their birth country [11]. In a recent cross-sectional and longitudinal study of a multi-generational Asian American cohort, emigrating to the U.S. was shown to reduce gut microbial diversity and function [12]. Alpha diversity was measured using the Shannon entropy, a quantitative index that accounts for the abundance and evenness of species residing in the host, as opposed to species richness, which is the number of species present. Within the gram-negative Bacteroidetes phylum, bacterial strains from the genus *Prevotella*, whose enzymes degrade plant fiber, became displaced by dominant strains from the genus *Bacteroides* according to an individual’s time spent in the U.S. The ratio of *Bacteroides* to *Prevotella* increased by factors of 10, correlating with the time in decades spent in the U.S. Prior to this study, metagenomics had identified three clusters of variation in the human gut, referred to as enterotypes [13]. The first enterotype, high in *Bacteroides* and low in *Prevotella,* is found in individuals on a long-term Western diet high in animal protein, the nutrient choline, and saturated fat [14]. The second enterotype is high in *Prevotella*, low in *Bacteroides*, and associated with a plant-based diet rich in fiber, simple sugars, and plant-derived compounds. While less distinct, a third potential enterotype was found with a slightly higher population of genus *Ruminococcus* within the phylum Firmicutes. Enriched *Ruminococcus* is associated with irritable bowel syndrome (IBS) [15], and transient blooms of pro-inflammatory *Ruminococcus* have been associated with active flare-ups in IBD [16]. *R. gnavus*, a prevalent gut microbe that proliferates in IBD, has been found to secrete a unique L-rhamnose oligosaccharide that induces tumor necrosis factor alpha (TNFα), a major pro-inflammatory cytokine [17].

## 2. Microbiota, the Immune Response, and Diet in IBD

IBD is a chronic GI disorder characterized by an overactive immune response to the gut microbiome. A serious, debilitating condition, IBD affects growth and development in children, increases the risk of colorectal cancer, and can lead to life-threatening complications [18]. There are two forms of IBD, Crohn’s disease and ulcerative colitis, that differ in the inflamed areas of the intestine. Normally, anaerobic microbes in the gut derive their nutrients from fermentation of indigestible oligosaccharides and other carbohydrates escaping proximal digestion [19]. In IBD, respiratory electron acceptors generated as a byproduct of the inflammatory host response become environmental stressors that support bacterial growth [20]. The disorder results in oxidative stress for the host and the microbiome, leading to gut dysbiosis in the form of decreased community richness and proliferation of facultative anaerobic Enterobacteriaceae and adherent invasive strains of *Escherichia coli* [16,20,21]. Drug therapies for IBD have traditionally included immunosuppressants in the form of corticosteroids, antimetabolite agents, or anti-TNF antibodies, often with ancillary administration of antibiotics [22]. An alternative treatment, given predominantly to children, is a defined enteral nutrition formula. Dietary therapy has the advantage of obviating the need for immunosuppression and is thought to work by altering the composition of gut microbiota.

A longitudinal study involving metagenomic analysis was conducted of 90 children initiating treatment for Crohn’s disease [22]. GI symptoms, mucosal inflammation, and microbial communities were compared for dietary and anti-TNF therapy and antibiotic use relative to healthy children. Microbial communities separated into two clusters based on composition. The dysbiotic community associated with active disease was characterized by increased fungal representation, increased lactose-fermenting bacteria *(Streptococcus, Lactobacillus, Klebsiella*), and the presence of human DNA in the stool (from epithelial cells and white blood cells). Crohn’s patients also had reduced relative abundance of *Prevotella* and increased *Escherichia* compared to healthy children. Treatment with antibiotics in the last six months was strongly associated with microbial dysbiosis [22], consistent with earlier findings that oral antibiotics for acne are a risk factor for new onset Crohn’s disease [23]. Antibiotic-treatment was observed to enrich fungi such as *Candida* and *Saccharomyces* [22]. Treatment with the enteral nutrition [24] or antibody therapies, on the other hand, reduced inflammation and markedly improved gut microbiota. The relative populations of fungi were reduced within one week of receiving the defined dietary formula, which lacked fiber [22]. Since a defined formula was effective for restoring healthy microbiota, it is conceivable that a more general oral diet with the proper nutrition can restore the intraluminal environment [25,26,27].

## 3. Microbial Metabolites and Short-Chain Fatty Acids

### 3.1. SCFA Receptor Activation

Short-chain fatty acids (SCFAs) have attracted considerable attention for their role in human health [28]. Obligate anaerobic bacteria (phyla Firmicutes and Bacteroidetes) encode a variety of enzymes for hydrolyzing complex carbohydrates (chains of sugar molecules) not digestible by the host such as resistant starch and fiber. Certain genera such as *Lactobacillus* and *Bifidobacterium* specialize in oligosaccharide fermentation, utilizing galactooligosaccharides (GOS), fructooligosaccharides (FOS), and polysaccharide inulin [29]. Carbohydrate fermentation by anaerobes provides the host with important SCFAs such as acetate, propionate, and butyrate [30]. Several receptors have been identified for SCFAs such as free fatty acid receptor 3 (FFAR3 or GPR41) and niacin receptor 1 (GPR109A) [31]. GPR41 and GPR109A are G-protein coupled receptors (GPCRs) found on intestinal epithelial cells, immune cells, and adipocytes. As endogenous agonists in GPCR signal transduction, SCFAs have a profound effect on physiological processes [32,33] independent of delivering calories to the host as carbon molecules [34]. GPR41 is associated with increased energy expenditure, leptin hormone expression, and decreased food intake [31,35]. Analogous to the activity of niacin, butyrate activates GPR109A to suppress colonic inflammation and colon cancer development [36]. Niacin is a known lipid-lowering agent: GPR109A inhibits triglyceride hydrolysis (lipolysis) in adipocytes, lowering blood levels of triglyceride and low-density lipoprotein (LDL) to reduce atherogenic activity. Acetate and propionate activate cell surface receptor GPR43 to induce neutrophil chemotaxis. GPR43 is anti-lipolysis and implicated in IBD, but contradictory results in mouse models leave doubt as to whether an agonist or antagonist will best treat colitis [35]. There is a growing interest in pursuing GPR41 and GPR43 as drug targets for the chronic inflammatory disorders asthma, arthritis, and obesity [37]. Much work remains to be done to establish the appropriate disease models needed to study these conditions.

Colonic epithelial cells (colonocytes) are the control switch separating microbial homeostasis from gut dysbiosis [38]. It is known that antibiotics deplete microbes that ferment essential SCFAs such as butyrate, which are normally responsible for maintaining microbial homeostasis [24,39]. The lack of butyrate silences metabolic signaling in the gut. Mitochondrial beta-oxidation in colonocytes becomes disabled, resulting in a transfer of oxygen, which freely diffuses across cell membranes from the blood to the GI lumen. Oxygen in the colon then allows for pathogenic facultative anaerobes such as *E. coli* [40] to outcompete the benign obligate anaerobes that characterize a healthy gut [41,42]. Microbial homeostasis is normally maintained by peroxisome proliferator-activated receptor gamma (PPAR-γ). PPAR-γ is a nuclear receptor activated by butyrate and other ligands, is found in adipocytes and colonocytes, and is responsible for activating genes involved in glucose and lipid metabolism. Lack of butyrate signaling results in nitrate electron acceptors being released into the colon, which facultative anaerobes can also use for cell respiration, breaking down carbohydrates into carbon dioxide rather than fermenting them [20]. Facultative anaerobes, including Proteobacteria, could further affect nutrition by catabolizing SCFAs present in the lumen [38]. The metabolic reprogramming of colonocytes is analogous to that of macrophages, which become polarized toward anaerobic glycolysis in response to proinflammatory signals. In ulcerative colitis, excessive epithelial repair results in lower PPAR-γ synthesis, which reduces beta-oxidation and increases oxygenation of colonocytes. Inflamed mucosae in colitis patients are increased in Proteobacteria, a major phylum of gram-negative bacteria, but decreased in gram-positive Firmicutes. Treatment with PPAR-γ agonist, however, can improve the microbial balance [43].

### 3.2. Fecal Biomarkers and IBS

Fecal biomarkers such as inflammatory proteins, antimicrobial peptides, and SCFA levels are emerging as a non-invasive screening tool for assessing and diagnosing various health conditions [44]. Patients with IBD have lower fecal levels of acetate, propionate and butyrate, and higher levels of lactic and pyruvic acids than healthy individuals [45]. Given the relationship between bacterial fermentation products and atherosclerosis, ongoing research aims to characterize the fecal microbiota and SCFA signatures of individuals with high blood lipid levels [46]. High levels of isobutyric acid could be one such biomarker for hypercholesterolemia. Colonoscopy is an invasive exam relied on in the United States as a periodic screen for colorectal cancer [18], but annual screening is performed in many countries using a non-invasive fecal immunochemical test, which looks for microscopic blood in the stool [47]. Current efforts are underway to identify novel microbial biomarkers for colorectal cancer given that it is associated with increased fecal levels of *F. nucleatum*, a promoter of tumorigenesis [3].

Unlike structural disorders such as IBD, IBS is a functional disorder and collection of GI symptoms observed in the absence of macroscopic signs of inflammation. Despite affecting 10–15% of the population and the potential for low quality of life, its etiology is unclear and current drug treatments are largely ineffective [48]. Diagnosis has traditionally relied on symptom criteria, stool characteristics, and questionnaires, once all other pathologies are ruled out [49]. The Rome criteria sets classifications for four subtypes: IBS with predominant diarrhea (IBS-D), IBS with predominant constipation (IBS-C), and IBS with mixed or alternating-type bowel habits (IBS-M) depending on whether >25% of bowel movements belong to soft or hard type stool categories or both, respectively, followed by IBS unclassified (IBS-U) [50]. It has been known for some time that IBS patients have reduced microbial diversity compared to healthy subjects [51], see also References 6–9 in [51]. Inflammatory proteins such as human β-defensin 2, a bactericide, have been identified as a useful fecal biomarker in IBS and IBD [48,51,52]. Lastly, the concentration difference in two SCFAs, propionic minus butyric acid, has been shown to be positive for all four IBS subtypes but negative in healthy subjects [53].

### 3.3. Leaky Gut

Elevated levels of interleukin 6, a pro-inflammatory peptide cytokine, and plasma levels of lipopolysaccharide (LPS) endotoxin, a marker of gram-negative bacterial translocation, were found to be elevated in a subpopulation of IBS-D patients with small intestinal permeability, analogous to that observed in celiac disease [54]. It is hypothesized that psychological stress can exacerbate the inflammatory condition by allowing translocation of harmful bacterial products across the intestinal epithelium. Known as “leaky gut”, a compromised epithelial barrier allows toxins and antigens in the GI lumen to enter the bloodstream. A healthy gut flora is important in maintaining the intestinal barrier. By increasing the expression of tight cell junction proteins, beneficial probiotics such as *Lactobacillus* and *Bifidobacterium* can limit the development of autoimmune diseases in genetically susceptible individuals [55] and fatty liver disease in obese individuals [56]. In alcoholic liver disease, alcohol consumption causes gut permeability by reducing the expression of REG3, a bactericidal protein normally responsible for restricting the mucosal colonization of luminal bacteria [57]. 

### 3.4. Gut-Brain Interactions

In the last decade, it has been discovered that the enteric and central nervous systems are linked via a bidirectional communication network termed the gut-brain axis. Gut-brain communication is disrupted in the cases of IBS and microbial dysbiosis [58], in the former leading to changes in intestinal motility and secretion and causing visceral hypersensitivity (hyperalgesia) [59]. Recurrent abdominal pain is a hallmark characteristic of IBS. Autism spectrum disorder, which is often associated with constipation, has been connected to gut dysbiosis in the form of an increased Firmicutes/Bacteroidetes ratio and high levels of facultative anaerobes *Escherichia/Shigella* and the fungal genus *Candida* [60,61]. It is suggested that leaky gut contributes to the pathogenesis of autism by increasing systemic metabolites that alter the neuroimmune and neuroendocrine systems, thus affecting the brain and neurodevelopment [61,62,63].

For the last century, the ketogenic diet (KD) has been used to treat refractory epilepsy in children’s hospitals [64], achieving a 50% reduction in seizure rates [65]. KD restricts the proportion of carbohydrate intake to create a state of ketosis in which the body relies on ketone bodies for energy rather than glucose. Clinical studies are now investigating the use of KD for treating neurological conditions including autism, Alzheimer’s, and Parkinson’s disease, with promising results obtained for small cohorts [66]. The mechanism of action was initially thought to result from the normalization of aberrant energy metabolism associated with these disorders, but the role of the gut microbiota is now coming into focus. A recent comparison of KD-fed conventionally raised mice versus mice treated with antibiotics or reared germ-free revealed that alterations in the gut microbiota are required to reproduce the anti-seizure effects of KD [67]. Following KD was observed to enrich the populations of the anaerobic genera *Akkermansia* and *Parabacteroides*. Moreover, increased levels of the inhibitory neurotransmitter γ-aminobutyric acid (GABA) were detected in metabolite profiles of the brain hippocampus of KD-fed mice and were observed to be microbiota-dependent. GABA is a principal means of reducing communication between brain cells, and neuronal excitability is enhanced in neurological conditions such as epilepsy, anxiety, and Alzheimer’s disease [66,68]. Besides dietary intervention, these and other observations suggest that supplementation with prebiotics or probiotics could be used to improve cognitive symptoms associated with neurological conditions ranging from autism to Alzheimer’s and Parkinson’s [69,70], giving rise to the notion of “psychobiotics” [71,72].

Fecal microbiota transplantation (FMT) is yet another therapeutic option, which involves the engraftment of microbes from a healthy donor [73]. In a study of 18 autistic children, an eight-week course of FMT resulted in behavioral improvement and an 80% reduction in GI symptoms and abdominal pain associated with autism [74]. Outcomes remained improved when assessed eight weeks after treatment had ended, lending support to the hypothesis that gut microbiota are at least partially responsible for autism symptoms. Analysis of microbiota composition showed that FMT increased overall bacterial diversity and the abundance of fermentative *Bifidobacterium* and *Prevotella* in autistic individuals even after treatment cessation. In other clinical studies, FMT has demonstrated a 90% success rate for treating recurrent *Clostridioides difficile* infection, clinical remission rates of up to 78% in treating IBD, and symptom resolution or improvement in up to 70% of IBS patients [75]. Interest is now growing for the application of FMT in other disorders ranging from Parkinson’s to metabolic syndrome [75,76]. In patients with metabolic syndrome, FMT was shown to improve insulin sensitivity for those with decreased baseline microbial diversity, but the effects did not persist in the long-term [77].

Other lines of clinical evidence on the gut-brain interaction show that gut microbiota influences the central nervous system by alterations in the release of neuroendocrine hormones and neurotransmitter activity. Dysfunctions in GABA receptor signaling are implicated in anxiety and depression, and beneficial bacteria *Lactobacillus* and *Bifidobacterium* convert the amino acid glutamate into GABA [78,79]. Metagenomic analysis of a 1054-person Flemish cohort revealed that butyrate-producing *Faecalibacterium* and *Coprococcus* associate with higher quality of life and improved mental health, while *Dialister* and *Coprococcus* are depleted in cases of depression [79]. To improve cognitive symptoms associated with clinical depression and anxiety, beneficial probiotic strains of *B. longum* and *L. helveticus* have been administered clinically with promising results [72,80]. In a study comparing young and middle-aged mice, dietary supplementation with prebiotic inulin was observed to increase *Bifidobacterium* and *Akkermansia,* reduce neuroinflammation and anxiety, and improve cognition in middle-aged mice [81]. The fact that alterations in gut microbiota can provide cognitive symptom relief could offer one basis for the relationship observed between quality of diet and one’s mental health status [82].

## 4. Gut Microbiota and Metabolic Syndrome

### 4.1. Obesity, Microbial Diversity, and SCFA Supplementation

Clear links are emerging between the microbiome and its effects on host metabolism, with profound implications for human health given the rise of obesity and metabolic syndrome in Western society [83]. A study of four twin pairs discordant for obesity by Ridaura et al. revealed differences in their microbiota [84], with the lean individuals exhibiting an increase in bacterial SCFA fermentation and transformation of bile acids. To show that SCFA production was transmissible, the human fecal microbiota was transplanted into lean and obese mice. Obese mice were also cohoused with lean mice for 10 days, which countered weight gain due to an invasion of their microbiome by specific members of Bacteroidetes when a low-fat diet was administered. Such findings highlight the role of environmental factors in shaping gut microbiota and the development of obesity.

A study of human and mouse microbiota correlated obesity with differences in the relative abundance of two dominant bacterial divisions and showed that obese individuals have an increased capacity to harvest energy from the diet [85]. Relative to lean mice and humans, obese individuals have an increased relative abundance of Firmicutes, and reduced abundance of Bacteroidetes. The observation that reduced microbial diversity enhances calorie harvesting is also supported by a metagenomic analysis comparing microbiotas belonging to identical and fraternal twins and their mothers [86]. More recent work demonstrated that individuals with low microbial gene count have more systemic inflammation, adiposity, insulin resistance, and dyslipidemia [87]. Low gene count individuals gained more weight over time and were dominant in *Bacteroides* and *Ruminococcus* genera, while 36 genera including *Faecalibacterium*, *Bifidobacterium*, *Lactobacillus*, and *Akkermansia* were significantly associated with high gene count, lean individuals. In an analogous study involving 49 overweight or obese individuals, following an energy-restricted diet for six weeks was observed to partially restore microbial gene richness [88].

In human and rodent studies, one species of the Verrucomicrobia phylum inversely correlates with obesity and T2D, *Akkermansia muciniphila*, a mucus colonizer that can use mucin as its sole carbon and nitrogen source in times of caloric restriction. Treatment in mice with a probiotic strain of *A. muciniphila* or its prebiotic FOS was shown to reverse high fat diet-induced weight gain and insulin resistance, increase intestinal endocannabinoids controlling inflammation and the gut barrier, and counteract diet-induced decreases in mucus layer thickness [89]. In mouse fed a high-fat/high-sucrose diet, polyphenol-rich cranberry extract was found to protect against metabolic syndrome and intestinal inflammation by increasing the relative abundance of *Akkermansia* [90]. In humans, *A. muciniphila* levels at baseline and after a six-week calorie restriction diet were observed to correlate inversely with fasting glucose, waist-to-hip ratio, and plasma triglycerides [91]. A recent pilot study was conducted in overweight or obese insulin-resistant volunteers. Daily oral supplementation with 10^10^ *A. muciniphila* cells was found to improve insulin sensitivity, reduce insulinemia, and decrease body weight over a three-month period [92]. Such successful studies suggest that *A. muciniphila* could find use as a next generation probiotic to combat metabolic syndrome [93].

Roux-en-Y gastric bypass (RYGB) surgery is one of the most effective treatments for morbid obesity and T2D. RYGB reduces adiposity, improves glucose metabolism, increases resting energy expenditure, and results in rapid and sustained weight loss, but these effects cannot simply be attributed to decreased food intake and absorption [94,95]. In patients post-gastric-bypass, the abundance of Firmicutes has been found to decrease [96]. *Prevotella* is observed to increase three months after surgery relative to obese individuals, while *Faecalibacterium prausnitzii* is lower in diabetic subjects and correlates negatively with low-grade inflammatory markers [97]. In a mouse model, RYGB has been shown to restructure microbiota via a rapid and sustained increase in the relative abundance of *Akkermansia* downstream of the site of surgery in the gut [94].

Jiao et al. examined the effects of orally administering doses of the SCFAs acetic, propionic, and butyric acid to weaned pigs [98]. SCFA administration was observed to decrease serum levels of triglycerides, total cholesterol, and insulin, while increasing serum concentrations of the leptin hormone. Remarkably, the study demonstrated that SCFAs attenuate fat deposition by inhibiting feed intake, reducing lipogenesis, and enhancing lipolysis. Another study of 12 men undergoing colonic infusions showed that receiving an enema containing SCFAs can increase fasting fat oxidation and resting energy expenditure [99]. In a healthy diet, the bacterial fermentation of fiber into SCFAs promotes microbial diversity and is one mechanism by which high fiber intake inhibits weight gain [100,101], even outweighing heritable contributions to obesity [102].

The metabolic effects of butyrate were measured in a study of mice fed a high-fat diet (60% of calories from lard) [103]. Oral but not intravenous administration of butyrate was shown to act on the gut-brain circuitry via the vagus nerve, decreasing food intake and preventing diet-induced obesity, hyperinsulinemia, hypertriglyceridemia, and fatty liver disease. Interestingly, butyrate also promoted fat oxidation and activated brown adipose tissue. The finding that butyrate improves energy metabolism without eliciting any ill effects suggests that oral supplementation might be a promising strategy for combatting cardiometabolic disease [104]. Butyrate was further shown to alter the gut microbiota independent of the vagus nerve [103]. Specific genera within the subclass Erysipelotrichia were significantly increased, bringing the relative abundance of the Firmicutes phylum from 26% to 32% relative to controls, while the Bacteroidetes phylum decreased from 71% to 66%. The ratio of Firmicutes to Bacteroidetes increased by 21% upon butyrate administration. Given that Firmicutes generally correlate with a less beneficial metabolic profile [105], it appears that specific species of Erysipelotrichia are beneficial to host energy metabolism.

### 4.2. Microbiota in Diabetes

Both obesity and diabetes are characterized by insulin resistance and low-grade inflammation. A mouse study by Cani et al. points to bacterial LPS as a causative factor of insulin resistance, obesity, and diabetes [106]. Feeding and fasting cycles increased or decreased plasma levels of LPS, respectively, and metabolic endotoxemia was observed in mice fed a four-week high-fat diet that increased the proportion of gram-negative bacteria in the gut, raising plasma LPS concentration by a factor of two to three. Endotoxemia could also be induced via subcutaneous infusion of LPS for four weeks, resulting in weight gain and increased fasting hyperglycemia and hyperinsulinemia. LPS produces inflammation in adipocytes through the activation of toll-like receptor 4 signaling [107]. Thus, prebiotics that improve intestinal microbiota and reduce intestinal permeability are of potential clinical use for the treatment of diabetes [108,109]. Randomized controlled trials have reported improvements in glycemia and cardiovascular markers in T2D patients taking resistant starch, resistant dextrin, or inulin [110]. 

Consumption of dietary fiber has positive metabolic health effects including increased satiety, decreased weight gain, and lowered blood glucose and cholesterol levels, serving to reduce the risk of CVD and T2D [111,112,113]. Fiber has historically been classified as either soluble or insoluble, but plant cell walls often contain both and this distinction does not always predict physiological function [114]. It can be more useful to classify fibers into four categories based on whether they are readily fermented and whether they form a viscous cross-linked gel [115]. Insoluble fiber (wheat bran) is poorly fermented and does not alter viscosity. Soluble, nonviscous fiber (inulin, wheat dextrin, resistant starch) is readily fermented. Conversely, viscous gel-forming fibers can be fermentable (β-glucan) or not (psyllium). Improvements in metabolism can arise from three factors: microbial fermentation of soluble fiber into SCFAs [33,95,100], delayed nutrient absorption and improved cholesterol/glucose due to viscous gel formation [115,116], and the ability of insoluble fiber to reduce insulin resistance by interfering with protein absorption [112]. In conventional rats, a high-fat diet was found to reduce butyrate formation and increase liver cholesterol and triglyceride content compared to rats fed a low-fat diet, but these effects could be partially reversed by adding fermentable dietary fiber to the high-fat diet [117]. In a 12-week mouse study, supplementing a high-fat diet with 10% fermentable flaxseed fiber dramatically increased butyrate production, energy expenditure, and *Bifidobacterium* and *Akkermansia* levels, while countering weight gain [118]. In contrast to the Western diet, consuming daily servings of fiber, fruit, and vegetables promotes the alpha diversity of bacterial species in the gut [12,102,119,120,121].

Suez et al. investigated the impact of non-caloric artificial sweeteners (NAS) on glucose tolerance [122]. Commercial formulations of saccharin, sucralose, or aspartame were added to the drinking water of lean mice for 11 weeks. The 10% NAS solutions were well below the known toxic doses given per kg body weight. While mice drinking water, glucose, or sucrose had similar glucose tolerance curves, all three NAS-consuming groups developed glucose intolerance, which could be reversed upon antibiotic treatment. NAS was also shown to induce changes in gut microbiota previously observed in T2D; notably, the over-representation of gram-negative *Bacteroides* and under-representation of gram-positive Clostridiales. Bacterial taxa were enriched in the metabolic pathways involved in glycan degradation, contributing to enhanced capacity for energy harvest [85]. Lastly, Suez et al. assessed long-term NAS consumption in a clinical nutrition study using a food frequency questionnaire given to 381 non-diabetic individuals. Significant positive correlations were found between NAS consumption and measures of metabolic syndrome including increased weight, waist-to-hip ratio, fasting blood glucose, and hemoglobin A1c [122].

The link between NAS consumption in mice and alterations in gut microbiota lends support to the notion that individuals can have a personalized response to dietary components based on existing or acquired differences in their microbiota. A study of 800 healthy and prediabetic Israelis revealed high interpersonal variability in their postprandial glucose responses to the same foods, which could be attributed to differences in gut microbiota and other factors [123]. A machine learning algorithm was developed by Zeevi et al. and found to accurately predict personalized glycemic responses to real-life meals using information on blood parameters, dietary habits, anthropometric measures, physical activity, and gut microbiota. Twenty-six new participants were then recruited for a randomized controlled trial. The algorithm was found to be capable of choosing a personalized diet that successfully lowered the post-meal glycemic responses for each individual [123]. An analogous study of Midwestern Americans predicted glycemic responses once the abundances of *Prevotella* and *Bacteroides* were taken into account [124]. Such studies highlight the significance of individual microbial profiles in constructing therapeutic interventions, of great potential relevance to the emerging field of personalized nutrition [125].

Finally, diabetes medications have been connected to positive changes in gut microbiota. Metagenomic analysis of 345 Chinese volunteers revealed that diabetics have a decrease in butyrate-producing bacteria and an increase in opportunistic pathogens relative to healthy subjects [126]. A four-month placebo-controlled study was recently performed on 40 newly diagnosed T2D patients [127]. In individuals given the gold standard T2D drug, metformin, rapid alterations were observed in the composition of the gut microbiome. In the entire cohort, a negative association was observed between hemoglobin A1c blood levels and *B. adolescentis*, a species whose replication rate was increased by metformin. Transfer of fecal samples before and after metformin treatment to germ-free mice showed that improved glucose tolerance can arise solely from the metformin-altered microbiota. At the chemical level, the antidiabetic effects were attributed to increased microbial production of SCFAs and changes observed in the bacterial expression of metal-binding proteins [127].

In a rodent study, mice fed a high-fat diet containing lard oil had reduced expression of sodium glucose cotransporter-1 (SGLT1) [128]. SGLT1 is normally required for healthy glucose sensing in the upper small intestine in order to lower endogenous glucose production by the liver. Treatment with metformin was observed to restore SGLT1 expression and enhance intestinal glucose uptake. Metformin also increased the abundance of *Lactobacillus* bacteria in the upper small intestine. The antidiabetic effect was transmissible upon fecal transplantation, showing that the intestinal microbiota restores SGLT1 expression and glucose sensing in untreated obese rats. Before treatment, mice consuming the high-fat diet had a decreased abundance of gram-positive phylum Actinobacteria, while phylum Proteobacteria and genus *Escherichia* were increased relative to the control group consuming regular chow. The molecular link to SGLT1 expression is unknown, but it is likely that microbial metabolites such as SCFAs activate glucose sensing. Metagenomic analysis of a Dutch cohort corroborated that SCFA concentrations are higher in metformin users compared to diabetics not taking metformin [120]. Analysis of a Colombian community found that metformin users had higher levels of SCFA-producing *A. muciniphila*, *B. bifidum*, and *Prevotella* [129].

A subset of patients cannot tolerate metformin due to adverse GI effects including abdominal pain, bloating, nausea, and diarrhea. A small clinical trial was recently conducted in nondiabetic individuals, confirming that metformin alters gut microbiota independent of glycemic status [130]. Interestingly, the bacterial abundance of 12 genera at baseline predicted whether healthy individuals would experience adverse GI effects upon treatment with metformin. This observation provides a glimpse at how gut microbiota, which are shaped by diet, can mediate individualized therapeutic responses to a medication. Lastly, diabetes medication acarbose is a minimally absorbed glucoamylase inhibitor that prevents starch digestion by humans. A mouse study monitored acarbose-treated mice fed either a Western-style high-starch diet or a high-fiber diet rich in plant polysaccharides [131]. Analogous to metformin treatment, high doses of acarbose were sufficient to alter gut bacterial taxa and increase butyrate production even in those consuming a high-starch diet, but the bacterial composition quickly reverted upon cessation of acarbose treatment. Altogether, these studies suggest that alterations in the gut microbial community are prominent contributors to the mechanism of action in antihyperglycemic agents.

### 4.3. Dietary Choline and Atherosclerosis

Metabolomic analysis was used to monitor 2000 metabolites present in the blood plasma of patients undergoing cardiac evaluation in order to identify potential predictors of CVD events [132]. Three small molecules were found to predict CVD risk: choline, trimethylamine *N*-oxide (TMAO), and betaine. Each are metabolites of phosphatidylcholine, a dietary lipid found in high quantities in egg yolk, liver, and other high-fat animal products. Choline, also called lecithin, is an essential nutrient that is marketed as a dietary supplement. Hydrolysis of phosphatidylcholine liberates choline, which is metabolized by gut microbes into trimethylamine (TMA) gas, which the liver in turn converts into TMAO. In mice fed radiolabeled phosphatidylcholine, increased blood levels of TMAO were revealed to contribute to greater arterial plaque development [132]. In another study, atherosclerosis susceptibility could be transmitted from atherogenic-prone mouse strains to atherogenic-resistant strains via cecal microbial transplantation [133].

The National Institutes of Health funded two prospective clinical studies on TMAO [134]. In the first study, the phosphatidylcholine challenge, plasma levels of TMAO were observed to rise after consumption of two eggs traced with isotope-labeled phosphatidylcholine. TMAO generation could be suppressed by administering a weeklong course of antibiotics to reduce gut bacteria. One month after withdrawal of antibiotics, TMAO generation returned in a follow-up choline challenge test. In a second cohort of 4007 adults undergoing cardiac evaluation, participants with the highest quartile of fasting plasma TMAO levels had a significantly increased risk of experiencing a major adverse CVD event within the three-year follow-up period (hazard ratio, 2.5, relative to lowest quartile). Another study of patients with stable coronary artery disease found a four-fold increase in all-cause five-year mortality risk for those in the highest TMAO quartile [135]. The atherogenicity of choline metabolite TMAO helps explain the correlation that exists between CVD and excessive consumption of animal products [136]. A causal link between dietary cholesterol and CVD, on the other hand, has not been demonstrated and would be difficult to prove given the fact that cholesterol-containing foods are also high in saturated fat, with the exception of eggs and shrimp [137]. A long-term study of 29,615 participants recently showed that consuming eggs with yolk elevates one’s CVD risk in a dose-dependent fashion [138], with each half an egg consumed per day elevating absolute risk by 1.1% and all-cause mortality by 1.9%. One egg yolk contains 120 mg choline.

A structural analog of choline and natural product found in some foods, 3,3-dimethyl-1-butanol (DMB), has been shown in mice to reduce TMAO levels by non-lethal inhibition of TMA lyase [139], giving credence to the notion of “drugging the microbiome.” In a study of mice fed a Western diet, DMB reduced plasma TMAO and prevented cardiac dysfunction, inflammation, and fibrosis, but had no effect on body weight and dyslipidemia [140]. Efforts are underway to determine the TMA-forming potential of different bacterial species and develop new treatment strategies for restraining the proliferation of TMA producers [141]. L-carnitine is another trimethylamine abundant in red meat that is also sold as a dietary supplement. Similar to choline, studies in rodents and humans show that carnitine increases plasma TMAO levels, accelerates atherosclerosis, and increases CVD risk [142]. Interestingly, comparison of carnitine challenge tests in habitual omnivores versus vegans/vegetarians reveals that omnivores harbor a microbiota capable of generating 20-fold higher levels of TMAO [142,143].

The connection between TMAO and CVD has important implications for meat consumption given that beef and pork contain 100 mg choline per 100-g serving (veal: 400 mg). Fish and chicken are not far behind with 70–80 mg choline per serving. Some studies have observed a modest increase in relative risk of CVD mortality (between 26% and 34%) for the highest quantile consumption of unprocessed red meat or both processed and unprocessed red meat [144,145]. Comparative risk assessment using a national survey, however, did not find a significant contribution for unprocessed red meat alone [146], and an earlier meta-analysis calculated its relative risk ratio per 100-g serving to be 1.00 (95% confidence interval: 0.81–1.23) [147]. It is likely that the quality of the comparison diet is a confounding variable contributing to disparate findings on the contribution of meat to CVD [148].

Improved cardiovascular health has been associated with one’s degree of adherence to a Mediterranean-style diet, which limits consumption of red meat and dairy while emphasizing plant-based foods and healthy fats [149,150,151]. The relative reduction in CVD morbidity risk obtained for those in the highest quantile of adherence to the Mediterranean diet, considering all dietary components combined, is observed from meta-analyses to be in the vicinity of 30%, or even up to 45% for high risk populations [152]. The microbiome was recently assessed by De Filippis et al. in 123 Italian individuals habitually following omnivore, vegetarian, or vegan diets [153]. To score their adherence to the Mediterranean diet, individuals were stratified along an 11-food unit dietary index. Individuals consuming vegetable-based diets had higher adherence to the Mediterranean diet, were increased in *Prevotella* and fiber-degrading bacteria, and had higher fecal levels of SCFAs. Omnivores on the other hand had a higher ratio of Firmicutes to Bacteroidetes in the gut and elevated TMAO in the urine [153].

The scientific community has also debated the extent to which red meat elevates the risk of colorectal cancer, another condition prominent in Western society [154,155]. Gut microbiota associated with colorectal cancer were recently shown to have an increase in genes associated with TMA lyase and protein catabolism, while microbe carbohydrate degradation pathways were depleted [156,157]. Dietary choline is not observed to correlate with cancer incidence, while betaine, a methyl group donor, is associated with reduced colorectal cancer risk [158]. Again, overall diet quality is likely a significant factor. A study using a polyposis cancer model in mice showed that a high-fiber diet increases SCFA-producing bacteria as well as the expression of butyrate receptor GPR109A, serving to suppress colon carcinogenesis [159]. A case-control study conducted in China found an inverse association between vegetable fiber intake and colorectal cancer (Q4 versus Q1 odds ratio: 0.51; 95% confidence interval: 0.31–0.85) [160]. Strong associations were also observed for total, soluble, and insoluble fiber intakes, but not fruit, soy, or grain fiber. A comparative risk assessment estimated that suboptimal food group intake levels account for 38% of new colorectal cancer cases [161]. Microbial overgrowth was recently shown to fulfill the ecological Koch’s postulates [162] of disease causation in colorectal cancer. Rather than a specific pathogen, a matrix-enclosed ecosystem of bacteria, or biofilm, extracted from tumor patients was found to induce tumorigenesis in mice [163].

## 5. Microbial Interventions

### 5.1. Probiotics

Probiotics are defined as “live microorganisms that, when administered in adequate amounts, confer a health benefit on the host” [164]. Probiotics are available over-the-counter or by prescription containing microorganisms similar to the commensal bacteria found in the gut, most commonly lactic acid-producing *Bifidobacterium* and *Lactobacillus* spp. As a whole, there is clinical evidence to support the use of probiotics for treating acute infectious diarrhea, antibiotic-associated diarrhea, *C. difficile*-associated diarrhea, ulcerative colitis, and irritable bowel syndrome, but not for acute pancreatitis or Crohn’s disease [165,166,167,168,169,170]. Commonly prescribed antibiotics carry a risk of *C. difficile* infection, which can cause severe complications and has an estimated treatment cost of $24,205 USD per patient. Co-administration of probiotics, which lower the risk of *C. difficile* infection, has therefore been proposed as a prophylactic whenever antibiotics are prescribed [171]. Clinical research into probiotics is species- and often strain-specific, with particular bacteria investigated for separate disease states [172]. Probiotic bacteria can potentially provide various health benefits through normalizing perturbed microbiota and intestinal motility, competitively excluding pathogens, and increasing SCFA production [173,174,175].

Different probiotic species have been studied for ameliorating GI symptoms, though it is not always clear which species or strains are most beneficial [176]. Earlier work observed that the ratio of Firmicutes to Bacteroidetes was elevated in 62 IBS patients relative to 46 control subjects in Helsinki, Finland [177]. Surprisingly, both groups were dominant in the relative abundance of Firmicutes (90% and 83%, respectively), leaving doubt as to the representativeness or overall health of the small cohort (64% was estimated for an 1135-person Dutch cohort [120]). *Bifidobacterium* was one genus of strictly anaerobic gram-positive Actinobacteria whose numbers were markedly decreased (16–47%) in patients diagnosed with IBS-M, IBS-D, or IBS-C relative to healthy controls [177]. Other studies have confirmed that probiotic supplementation with bifidobacteria results in modest improvement of GI symptoms experienced in IBS-C and IBD patients [167,178]. Correlating microbial profiles to gut health is more complicated for other species. Within the Firmicutes phyla, *Streptococcus* are found to be decreased in IBS-C but increased in IBS-D, while *Allisonella* are decreased in IBS-C and IBS-D but increased in IBS-M [15]. Genera within Bacteroidetes such as *Prevotella* and *Bacteroides* may be increased or decreased in IBS [15,177]. It has been noted that there is a strong positive association between IBS and small intestinal bacterial overgrowth (SIBO) [179]. This gave rise to the initial idea of treating the condition with antibiotics, but patient response varies widely and GI symptoms may even worsen. Recent antibiotic exposure actually correlates positively with the development of SIBO [180]. SIBO and GI symptoms have been shown to be exacerbated in healthy individuals who switch to a high-sugar, low-fiber diet for only seven days, leading to a decrease in small intestinal microbial diversity and an increase in epithelial permeability [180].

One challenge with the probiotic market is that, unless specific disease-related claims are made, commercial products are poorly regulated. Probiotics are trademarked by brand rather than by bacterial strain, and formulations or manufacturing protocols can change over time, having a dramatic impact on efficacy [181]. It has been shown in particular that strains within the same genus or species can have substantially different effects on the host, differing in their ability to grow and survive the intestinal environment, adhere to intestinal epithelial cells, and inhibit pathogen invasion [182,183]. After the isolation of *E. coli* Nissle 1917 from the stool of a World War I soldier who did not catch dysentery, nonpathogenic strains of *E. coli* gained some acceptance as probiotics. *E. coli* is unique in that it relies on monosaccharide and disaccharide nutrients broken down from complex carbohydrates by strict anaerobe species of bacteria [184]. Beneficial *E. coli* strains have been used to treat patients suffering from infectious diseases, likely due to their ability to outcompete enteric pathogens for nutrients [40]. Recent mouse studies give cause for caution, however. Cocolonization of *E. coli* O157:H7, a notorious foodborne pathogen, with a nonpathogenic strain of *E. coli* in germ-free mice actually increased the pathogen’s virulence and production of Shiga toxins, which are encoded by viral prophage genes, by up to 12-fold [185]. In another study, probiotic *E. coli* Nissle 1917 was observed to undergo genomic adaptation in response to selective and diet-dependent host pressures within a transit period of five weeks [186] To gain advantage especially in low-diversity guts, competitive adaptations in genes were acquired that affected intestinal adhesion and the utilization of carbohydrates and mucin components as carbon energy sources. In mice that were previously exposed to antibiotics, the *E. coli* strains acquired mutations responsible for antibiotic resistance [186]. Such studies underscore the centrally important role that horizontal gene exchange plays in the evolution of gut bacteria [187].

Several species of *Lactobacillus* and *Bifidobacterium* have now become the staples in the field of probiotics. Notable commercial multi-strain formulations have been subjected to clinical studies including Visbiome^®^ (formerly VSL#3) [188], BIO-25 [189], and Ther-Biotic^®^ Complete [190]. Visbiome^®^ contains several strains from well-known probiotic species *L. plantarum* DSM24730, *Streptococcus thermophilus* DSM24731, *B. breve* DSM24732, *L. paracasei* DSM24733, *L. delbrueckii subsp. bulgaricus* DSM24734, *L. acidophilus* DSM24735, *B. longum* DSM24736, and *B. infantis* DSM24737. Lactobacilli and bifidobacteria such as these have been extensively tested for their anti-inflammatory effects in colitis as well as their beneficial effects on gut motility, particularly for the treatment of constipation [173,191,192,193]. While *E. coli* is LPS-producing, *B. breve* has been shown to reduce LPS-induced epithelial cell shedding, which is observed in relapsing IBD patients [194]. Populations of *Lactobacillus* are reduced in alcohol consumption and in high fat diet-induced obesity [55,195]. Supplementation with probiotic strain *L. rhamnosus* GG has been shown to decrease microbial overgrowth, restore mucosal integrity, reduce microbial translocation, and ameliorate alcohol-induced liver injury [55,196]. Lastly, the use of probiotics has been proposed as an alternative or adjuvant to antibiotic treatment [197]. In the case of enterohemorrhagic *E. coli* O157:H7, antibiotics are not effective due to the release of additional toxin. Probiotics *L. acidophilus* R0052 and *L. rhamnosus* R0011 have been observed to prevent epithelial injury by reducing adhesion of *E. coli* O157:H7 and also enteropathogenic *E. coli* O127:H6 [198]. 

A clinical study of healthy adults given the probiotic *L. paracasei* DG revealed that the changes observed in the underlying gut microbiota can depend on an individual’s starting microbial profile [199]. Study participants with low initial fecal butyrate levels experienced a four-fold increase in butyrate production and a 55% decrease in *Ruminococcus*, a member of the Clostridia class responsible for degrading resistant starch. On the other hand, individuals with high starting butyrate levels experienced a 49% decrease in butyrate production and a decrease in six Clostridia genera including *Faecalibacterium*, an anti-inflammatory butyrate producer beneficial to mental health [79]. Other studies corroborate that a patient’s initial fecal microbial pattern can help predict their response to a probiotic intervention [189], suggesting it will one day be possible to optimize the dose of bacterial strains administered for an individual [200]. An individual’s microbiome has also been shown to influence the production of butyrate upon dietary supplementation with fermentable resistant starch according to which bacterial taxa become amplified [201]. Given the relation between the microbiome and metabolic disease, current research is now exploring probiotic interventions as an adjuvant therapy for improving cardiometabolic profiles [202,203]. Positive results have been obtained using the multi-strain formulation Ecologic^®^ Barrier for T2D [204]. In rats, Ecologic^®^ Barrier was previously shown to improve depression-related behavior independent of consumption of a high-fat Western-style diet [205]. Ecologic^®^ Barrier contains the following strains: *Bifidobacterium bifidum* W23, *B. lactis* W52, *Lactobacillus acidophilus* W37, *L. brevis* W63, *L. casei* W56, *L. salivarius* W24, *Lactococcus lactis* W19, and *Lc. lactis* W58. Lastly, two strains of *L. gasseri* isolated from human intestine and breast milk were found to reduce visceral fat mass in obese adults, but the effects diminished once treatment with SBT2055 was ceased, indicating that the probiotic needs to be continually supplied [206,207]. 

### 5.2. Prebiotics

In some clinical studies, a probiotic is administered in combination with a prebiotic compound that promotes bacterial growth, together termed a synbiotic. The requirements of a prebiotic are that it is not digested in the upper GI tract, can be fermented by intestinal microbiota, selectively stimulates beneficial bacteria growth and diversity, and has a positive effect on host health [208,209]. Prebiotics include FOS, GOS, and polyol sugar alcohols used as nutritive sweeteners [193,210]. Inulin is a soluble fiber and fructan, or variable length polymer of fructose, that is indigestible to humans and has minimal impact on blood glucose levels [211]. Believed to be most effective in nurturing the growth of many species of probiotic [193], inulin has been tested in successful synbiotic treatments for ulcerative colitis [191,211]. More recently, supplementation with butyrate and inulin was found to lower diastolic blood pressure, fasting blood sugar, and waist-to-hip ratio in T2D patients [104].

Numerous studies reveal that significant health benefits can be obtained from prebiotic administration alone [110,193,211]. Prebiotics such as GOS and FOS have been shown to improve microbial profiles by increasing bifidobacteria and decreasing *E. coli* [193,212]. See Table 5 in Reference [193] for a summary of prebiotic clinical trials. In a double-blind, randomized controlled trial of two separate cohorts in Canada, 16 weeks of FOS-enriched inulin supplementation (8 g/day) decreased body fat, serum triglycerides, and interleukin 6 in overweight or obese children compared to those given an isocaloric dose of maltodextrin placebo [213]. Bifidobacteria in fecal samples increased from 6% to 10% of mean bacterial abundance with prebiotic treatment, while Firmicutes decreased from 69% to 63% and *Ruminococcus* from 2.3% to 1.4%. In an animal study, rats fed a high-fat/high-sucrose diet along with FOS experienced a normalization in insulin resistance, leptin levels, dyslipidemia, and gut microbiota [214]. Moreover, prebiotic FOS was observed to limit knee joint damage in this diet-induced model of osteoarthritis, to levels approaching that obtained with moderate aerobic exercise. The effects of prebiotic therapy also depend on individual’s starting microbial profile. In a study comparing FOS, sorghum and arabinoxylan, equally high SCFA production was observed in volunteers whose microbiota was dominant in fiber-utilizing *Prevotella*, but *Bacteroides*-dominated individuals showed different SCFA levels in response to each fiber [215]. 

Given the relationship between gut microbiota and inflammation, research is underway to examine the effects of anti-inflammatory omega-3 polyunsaturated fatty acids (PUFAs) on microbial diversity. Consuming a Western diet high in animal protein is known to elevate the ratio of omega-6 to omega-3 PUFAs by up to a factor of 10, producing an inflammatory response mediated by hormone-like eicosanoids in the body [149,216]. The omega-3 PUFAs docosahexaenoic acid (DHA) and eicosapentaenoic acid (EPA), however, are inflammation-resolving and have anti-colorectal cancer activity, see References 4–6 in [217]. Human studies show that dietary supplementation with EPA and DHA increases the intestinal abundance of *Bifidobacterium* and *Lactobacillus*, while decreasing *Faecalibacterium* [217,218]. Conflicting results were reported for the effect of omega-3 fatty acids on the ratio of Firmicutes to Bacteroidetes phyla. Lastly, a metabolomic analysis was recently conducted of 876 adult female twins. After adjusting for dietary fiber intake, the consumption and circulating levels of omega-3 fatty acids were found to be significantly correlated with microbial alpha diversity as measured by the Shannon index [219].

## 6. Implications for Diet and Nutrition

### 6.1. Dietary and other Microbiome Covariates

A metagenomic analysis was conducted of 1135 participants from a Dutch population using deep sequencing [120]. The sequencing data enabled the detection of associations in microbiota with 126 different environmental factors including diet, disease, and medication use. Higher intakes of total carbohydrates were most strongly associated with decreased microbiome diversity: bifidobacteria increased while *Lactobacillus, Streptococcus,* and *Roseburia* genera decreased. The Shannon diversity index decreased according to intake levels of total carbohydrates, followed by sugar-sweetened beverages, bread, beer, savory snacks, and, to a lesser extent, total fats, pulses, and legumes. Diversity was also reduced in individuals self-reporting IBS, and antibiotic use was associated with decreases in two species of *Bifidobacterium*. On the other hand, microbial diversity increased with fruit, coffee, vegetable, and red wine intake and to a smaller extent eating breakfast and drinking tea. Red wine consumption was associated with an increased abundance of *F. prausnitzii* [120], an anti-inflammatory species implicated in lean-type, high-richness microbiota [87]. Coffee, tea, and red wine are high in polyphenols, compounds associated with prebiotic and bifidogenic activity, see References 19–21 in Reference [120]. In a recent meta-analysis, consuming up to three cups/day in coffee was found to decrease all-cause and CVD mortality in a dose-dependent fashion irrespective of caffeine content [220].

A similar population-level analysis of an 1106-person Belgian cohort across 69 covariates [221] showed that the Bristol stool scale, an indicator of gut transit time, and the use of medications have the largest explanatory value for microbiome variation. A total bacterial richness of 664 genera was found, but variance between individuals arose primarily from differences in the relative abundance of 14 core genera. Consistent with previously characterized enterotypes [13], bacterial taxa with the largest variation in abundance were *Prevotella*, *Bacteroides*, and Ruminococcaceae. *Prevotella* correlated with softer type stools, while Ruminococcaceae was the dominant family in hard type stools. Overall species richness declines with shorter gut transit times and the abundance of core species increases, likely because specific bacteria are selected for with a fast growth potential or high degree of mucosal adherence to avoid washout [221,222]. Other factors that turned out to be microbiome covariates were recent smoking history as well as the use of antibiotics, osmotic laxatives, IBD drugs, and antidepressants [221]. In a recent mouse study, six days of treatment with over-the-counter laxative polyethylene glycol had long-term effects on the gut [223]. Bacterial family S24-7 went from 50% of total microbial abundance to apparent extinction, while family Bacteroidaceae, also in order Bacteroidales, experienced an expansion from 20% to 60% microbial abundance. Osmotic stress was observed to decimate the mucus barrier and cause the immune system to generate a lasting antibody response against commensal bacteria [223]. Fecal samples were recently collected from 758 Korean men to examine the effects of cigarette smoking on the microbiome [224]. While no differences were observed between former smokers and those who never smoked, current smokers had an increased proportion of Bacteroidetes and decreased levels of Firmicutes and Proteobacteria.

Notable dietary covariates in the Belgian population study included consumption of fruits, alcohol, meat, soy products, and soda as well as one’s preference for dark chocolate [221]. Surprisingly, mode of birth and history of breastfeeding were not associated with one’s adult microbiota composition, and household pets only predicted a minimal fraction of microbiome variation [221]. An earlier study showed that household dogs primarily alter their owner’s skin microbiota rather than the gut microbiota [225]. More dominant influencers of the microbiome are the urbanization of outdoor areas, increased building confinement, and cleaning, each of which diminish overall microbial diversity, shifting from gram-positive (e.g., Actinobacteria) to gram-negative and potentially pathogenic species [226,227,228].

Consistent with the Belgian [221] and other studies [7,120], earlier analysis of the Dutch population cohort revealed that bacterial taxa could explain BMI and blood lipids independent of age, gender, and host genetics [229]. Species richness was negatively correlated with both BMI and triglycerides and positively correlated with protective levels of HDL cholesterol [120,229]. A significant correlation is not observed, however, between gut microbiota and LDL or total cholesterol levels [7,120,221,229]. The absence of correlation between plasma LDL and the microbiome is notable given that the latter is associated with metabolic disease. Despite plasma LDL being used as the principal target in lipid-lowering therapy for the last three decades, recent evidence suggests that triglyceride, HDL, and apolipoprotein B blood levels may be more useful CVD predictors [230,231,232,233,234,235]. Many factors confound the relationship between plasma LDL concentration and CVD. While one in three individuals are hyper-responders to dietary cholesterol, the ratio of LDL to HDL is minimally affected when others, particularly the elderly, consume an additional 100 mg/day [236]. For individuals with similar LDL concentrations, a predominance of small dense LDL particles (sdLDL) increases one’s CVD risk [236], as does a higher proportion of covalently modified LDL particles, known as lipoprotein(a) [237]. Widely prescribed statin drugs are effective at lowering LDL and to some extent apolipoprotein B concentration, but they do not decrease the proportion of sdLDL and have been found to raise plasma lipoprotein (a) by up to 20%, contributing to what has been termed “residual” CVD risk [238,239]. The lack of an association between plasma LDL concentration and the microbiome is not surprising given these confounding factors.

### 6.2. FODMAPs and Gut Health

Fermentable oligosaccharides (fructans, GOS), disaccharides (lactose), monosaccharides (fructose), and polyols (sorbitol, xylitol) are termed FODMAPs [240]. Consumption of dietary FODMAPs pulls water into the small intestine and colon, causing luminal distension. Fermentation of FODMAPs by gut bacteria and yeast then produces hydrogen or methane gas. Restricting FODMAPs in one’s diet has been shown to help alleviate functional GI symptoms in IBS patients (bloating, abdominal pain, diarrhea), but no effects have been reported for intestinal inflammation in IBD [27,240]. Wheat, rye and barley contain fructans and supply much of the FODMAPs contained in the Western diet. A double-blind crossover challenge was conducted of 59 adults self-reporting non-celiac gluten sensitivity (NCGS), who had previously followed a gluten-free diet for at least six months [241]. Participants completed three seven-day challenges in which a muesli bar was consumed containing either FOS, wheat gluten, or placebo, with the amounts of fructan/gluten equal to that contained in four slices of wheat sandwich bread. IBS symptom scores worsened in the fructan challenge (*P* = 0.04), while symptoms were actually slightly improved relative to placebo upon consumption of gluten (*P* = 0.55). The finding that fructans are responsible for GI symptoms in self-identified NCGS patients, and not gluten, is also supported by a crossover trial in which 37 subjects with NCGS and IBS followed a low-FODMAP diet before switching to a high- or low-gluten diet [242]. Regardless of the source of symptoms, NCGS and IBS at least have overlapping features and are not entirely separate entities [243].

Long-term implementation of a low-FODMAP diet is problematic due to the restriction of healthy plant foods and the fact that FODMAPs are prebiotics that support gut microbiota. Apples, pears, and stone fruits are high in fructose and other FODMAPs. Legumes and pulses are also high FODMAP, as are several vegetables including onion, garlic, and cauliflower. When administered properly by a trained dietitian, the FODMAP elimination diet is intended to be a process rather than a rigid exclusion diet. The initial elimination phase lasts 2–6 weeks in order to get GI symptoms under control. In the challenge phase, specific foods or types of FODMAPs are reintroduced one at a time and in increasing amounts. The patient is instructed to keep a detailed food diary so they can learn what FODMAPs are best tolerated and can eventually be incorporated into the final integration phase of the diet. Two clinical challenges can occur during this process: a patient’s symptoms may not respond, or they do respond and then the patient becomes reluctant to reintroduce FODMAPs [244]. While long-term studies are lacking, following a low-FODMAP diet reduces the diversity and quality of dietary components being consumed [245], and healthy diet diversity has been linked to more diverse microbiota and better health outcomes [246]. Short-term FODMAP restriction has been shown to disturb the gut microbiota in as little as 2-3 weeks, reducing total bacterial abundance and the population of *Bifidobacterium*, while increasing the ratio of Firmicutes to Bacteroidetes [247,248].

### 6.3. Ketogenic Diet

KD and low-carbohydrate diets have become a popular and effective tool for losing weight and can improve blood CVD parameters in the short-term [249,250]. However, 20-year studies involving a large prospective cohort reveal that diet quality and the source of protein and fat can ultimately determine health outcomes in low (40% of caloric intake) carbohydrate diets [251,252]. In research by de Koning et al., it was found that high plant-based intake of protein and fat reduces the hazard ratio (HR) for T2D to 0.78, whereas high intake of animal protein and fat maximizes the risk (HR: 1.37) [251]. Adjusting for red and processed meat intake was observed to lower the association with animal sources (HR: 1.11). In strict KD, below ground vegetables and legumes high in net carbs, and most fruits, are restricted in order limit total carbohydrate intake to 50 g/day. Restricting plant-based carbohydrates can have considerable effects on gut microbiota given that fiber and prebiotics are required for bacterial diversity [65,119,208]. The reduction in fiber can also contribute to constipation, a common side effect of KD.

In an anti-seizure mouse model, KD was shown to reduce gut bacterial alpha diversity, while elevating the relative abundance of *A. muciniphila*, but KD was only followed for three weeks [67]. A much longer study of 10 multiple sclerosis patients found that total bacterial abundance and diversity decreased in the short-term but recovered during weeks 12–24 of KD treatment [253]. *Akkermansia* was observed to increase initially but then declined during long-term KD and pioneer bacteria steadily declined [253]. Pioneer bacteria such as *Bifidobacterium* and *Clostridium* are the first to colonize newborns and patients recovering from a course of antibiotic treatment. Twenty children with refractory epilepsy were recently treated with KD for six months [254]. Treatment lowered alpha diversity and decreased the Firmicutes/Bacteroidetes ratio. In 10 of the children who were non-responsive to treatment (<50% seizure reduction), the relative abundance of Ruminococcaceae and Clostridia became enriched, suggesting specific bacteria may serve as an efficacy biomarker or potential therapeutic target [254]. Such alterations in gut microbiota associated with long-term KD suggest the importance of a properly balanced, high quality diet [65].

### 6.4. Role of Carbohydrate Intake

Consuming excess carbohydrates as part of a Western diet high in refined grains, starch, and added sugar negatively impacts gut microbiota. The first connection between the microbiome and metabolic health was noted in 1970, when the International Sugar Research Foundation found that a high-sugar diet led to high serum triglycerides in conventional rats but not germ-free rats [255]. In a modern Dutch population study, the largest dietary predictor of low gut bacterial diversity was the total intake of carbohydrates, followed by consumption levels of beer, bread, and soda [120]. A study of 178 elderly subjects by Claesson et al. found that patients in long-term residential care consumed a diet higher in fat and lower in fiber than seniors living in their community [246]. Diet diversity was scored using the healthy food diversity index, which differentiates between healthy and unhealthy foods across all food groups, and found to positively correlate with gut bacterial diversity. Individual microbiota clustered based on long-term care or community living status, and microbiota composition significantly correlated with frailty, co-morbidity, and inflammation markers [246]. While obesity research has traditionally compared low versus high fat diets, a rat study found that a low-fat/high-sucrose diet led to reduced bacterial diversity, increased Firmicutes: Bacteroidetes, a bloom in Ruminococcaceae, gut inflammation, altered vagal gut-brain communication, and obesity, similar to an isocaloric high-fat/high-sucrose diet [105].

Diets high in total carbohydrates and sugar correlate with increased fungus *Candida* and methanogen *Methanobrevibacter*, genera from different domains of life that correlate negatively with consumption of amino acids, protein, and fatty acids [256]. *Methanobrevibacter smithii* is the most prevalent archaeon in the human gut and can comprise up to 10% of all anaerobes in healthy adults. In a mouse model, *M. smithii* has been shown to increase host adiposity by directing *Bacteroides thetaiotaomicron* to ferment plant polysaccharides (fructans) in the diet to the SCFA acetate [257]. Bacterial fermentation of undigested dietary polysaccharides into SCFAs is estimated to account for 5 to 10% of daily caloric intake in the typical diet [258]. Elevated *M. smithii* has also been identified in IBS patients, especially those with IBS-C, in whom methane gas delays gut transit [259]. *M. smithii* copy number was observed to correlate inversely with stool frequency (*R* = −0.42).

*Candida* are the predominant fungal species capable of colonizing the gut. Overall the mycobiome is less stable than the microbiome [260]. While bacterial population structure primarily associates with long-term diet [14,246], *Candida* can vary extensively in time in response to recent carbohydrate consumption, antibiotic use, and environmental sources [22]. In a study of 98 healthy volunteers by Hoffmann et al., *Candida* correlated positively with long-term intake of total carbohydrates and sugar, and was strongly associated with recent carbohydrate intake [256]. Unlike *Candida* and *Methanobrevibacter*, bacterial populations were observed to associate more strongly with long-term dietary habits than with recent food consumption. *Prevotella* and *Ruminococcus* increased with carbohydrate intake and decreased with animal products, while the reverse effect was observed for *Bacteroides* [256]. A model of syntrophy was proposed in which methanogenesis supports *Ruminococcus* metabolism and *Candida* degrades starch into simple sugars, allowing for substrate fermentation by *Prevotella*. 

Stool sample studies have found *Candida* in 63% of individuals, with 11% showing *Candida* overgrowth [261]. Overgrowth can lead to invasive, systemic fungal infection in cancer patients or immunocompromised individuals, resulting in a high mortality rate. In a mouse chemotherapy model, *C. albicans* infection was observed to drive mucosal dysbiosis, allowing *Stenotrophomonas*, *Alphaproteobacteria*, and lactic acid-fermenting *Enterococcus* to proliferate while bacterial diversity declined [262]. Antibiotic treatment is also a strong risk factor for systemic candidiasis. In cell growth assays, SCFAs and lactic acid are shown to have a fungistatic but not fungicidal effect, suggesting that a healthy microbiome prevents *Candida* overgrowth [263]. Lactic acid is responsible for the antimicrobial activity of lactobacilli towards pathogens. Beneficial probiotic strain *L. rhamnosus* GG was additionally shown to bear an exopolysaccharide that interferes with *Candida* growth, hypha formation, and intestinal adhesion [264].

Excessive sugar or starch consumption can lead to *Candida* dysbiosis. Candidiasis is mostly attributed to *C. albicans*, a species which has intrinsic resistance to the fungistatic effect of SCFAs. Interestingly, SCFA resistance is dependent on monomeric glucose being present in the growth media; growth rates are attenuated when the disaccharide maltose is used as a nutrient source [263]. In a study of 120 individuals with chronic intestinal *Candida* overgrowth, diet therapy cured 85% of patients three months after conventional antifungal therapy, compared to 42% of subjects receiving nystatin alone [261]. Patients in the diet group avoided foods high in simple sugars and starch, cured and fatty meats, milk and dairy products, and alcohol.

The notion of cutting starch and sugar to promote intestinal health can be traced to the 1920s, when gastroenterologist Sydney Haas began treating celiac patients using the specific carbohydrate diet (SCD) [265]. SCD was later popularized as a diet for reducing microbial overgrowth by biochemist Elaine Gottschall, who created a dictionary of legal/illegal foods and ingredients [266,267]. The diet prohibits grains (wheat, barley, oats, rice, corn), potatoes, processed meats, added sugars, and disaccharides (lactose, sucrose), while allowing fresh (not canned) fruit, vegetables, and juices not from concentrate [268]. SCD limits dairy to butter, eggs, and aged cheeses containing minimal lactose. Beer, sweet wine, liqueurs, and mucilaginous fibers are restricted as are additives and preservatives like maltodextrin, pectin, guar/gums, and FOS. Sugar alcohols are prohibited, and honey is the recommended sweetener in SCD. A strict three-month period is first observed to starve off overgrowing bacteria and yeast, after which legumes may be selectively introduced. Unlike a low-FODMAP dietary strategy, SCD is intended to be a long-term exclusion diet. While avoiding FODMAPs can improve IBS symptoms in the short-term, cases of drug-free clinical remission have been reported in IBD patients following SCD, with complete resolution of mucosal inflammation in some Crohn’s patients [27,269].

Artificial food ingredients are specifically being linked to gut dysbiosis. Maltodextrin, a polysaccharide derived from starch hydrolysis, is a common food additive that enables adherent invasive strains of *E. coli* to adhere to intestinal epithelial cells and grow into biofilm, contributing to gut dysbiosis and intestinal inflammation [21]. Polysorbate-80, an emulsifier used in processed foods, has been shown to enhance translocation of pathogenic *E. coli* strains across colonocytes [21]. In a mouse study by Chassaing et al., low (0.1–1.0%) mass concentrations of emulsifiers polysorbate-80 and carboxymethylcellulose induced low-grade inflammation, obesity, and dysglycemia in wild-type mice and promoted robust colitis in mice predisposed to the disorder [270]. Fecal transplants to germ-free mice demonstrated that changes in microbiota were responsible. The emulsifiers reduced microbial diversity and levels of health-promoting Bacteroidales, while increasing mucolytic *Ruminococcus gnavus* and pro-inflammatory Proteobacteria. Reduced mucus thickness was also observed in the emulsifier-treated mice, along with bacterial encroachment into the normally sterile inner mucus layer [270]. Microbiota encroachment has been implicated in IBD and metabolic syndrome. In humans, the average bacterial-epithelial distance of closest bacteria correlates inversely with BMI, fasting glucose levels, and hemoglobin A1c [271]. Such observations point to the consumption of processed foods as one potentiator of the global obesity epidemic [272].

### 6.5. Intermittent Fasting

Excessive caloric intake results in fat being stored in white adipose tissue, while energy expenditure by fat oxidation predominantly occurs from thermogenesis of brown adipose tissue. Conversion of white adipocytes, known as beiging, is thus a promising strategy for treatment of metabolic disease. Recently, Li et al. were able to selectively induce the beiging of white adipose tissue in mice using the natural strategy of intermittent fasting [273]. Mice placed on an every-other-day fasting regimen had the same cumulative food intake as the ad libitum control group, but experienced a shift in gut microbiota, increase in fermentation products acetate and lactate, and a reversal of diet-induced obesity. Transport of acetate and lactate across the adipocyte membrane is driven by monocarboxylate transporter 1, whose expression was found to be upregulated in beige cells. Beiging was not observed in germ-free mice, but could be restored upon fecal transplantation of gut microbiota [273]. A previous study in mice demonstrated that cold exposure activates white fat beiging and increases insulin sensitivity via changes in the microbiome [274]. These observations reveal the existence of a microbiota-beige fat axis. In other work, Panda et al. found that diet-induced obesity dampens daily cyclical fluctuations in mice microbiota [275]. Restricting feeding to an eight-hour window each day partially restored circadian fluctuations, including a decrease in the abundance of *Lactobacillus* observed during the feeding phase. Intermittent fasting, longer multiday fasts, and fasting-mimicking diets have been shown to improve gut barrier function, increase microbial diversity, enhance antioxidative microbial pathways, and even reverse intestinal inflammation in models of IBD [276,277,278].

## 7. Other Considerations

### 7.1. Endocannabinoid System

In addition to altered microbiota and low-grade inflammation, obesity is characterized by increased endocannabinoid (eCB) system tone. A study of the eCB system in lean and obese mice was performed by blocking or activating cannabinoid receptor 1 (CB_1_) [279]. SR141716A, a CB_1_ antagonist that reduces food intake, significantly reduced gut permeability and plasma LPS levels in obese mice, decreasing both adiposity and blood glucose levels. In contrast, agonist HU-210 increased eCB system tone in lean mice and raised plasma LPS. Increased gut permeability with HU-210 was attributed to a decrease in the expression of two epithelial tight junction proteins. By comparing diet-induced obesity and intervention with antibiotics or prebiotics, microbiota associated with obesity were shown to be responsible for increasing the expression levels of CB_1_ in colonocytes and adipose tissue [279].

Endocannabinoids are an appealing therapeutic strategy for many conditions such as treating inflammation in IBD [280]. Cannabinoid antagonist cannabidiol has been shown to counteract the inflammatory environment induced by LPS in mice and in human colonic cultures derived from ulcerative colitis patients, at least in part due to PPAR-γ activation [281]. The use of CB_1_ agonists has been proposed for increasing GI transit time in IBS-D, while antagonists could prove useful for IBS-C [282]. Partial agonist tetrahydrocannabinol (THC) increases food intake in the short-term, but in epidemiological surveys, obesity is observed to be less prevalent among cannabis users [283]. In mice fed a high-fat diet, chronic treatment with THC was recently shown to stave off increases in the ratio of Firmicutes to Bacteroidetes, increase the abundance of *A. muciniphila*, and prevent diet-induced obesity [284]. 

### 7.2. Medication Dysbiosis

Oral administration of high dose antibiotics can result in rapid changes to gut microbiota and is implicated in dysbiosis [22,285,286,287]. Over-the-counter and prescription non-antibiotic medicines also influence the gut microbiome. Proton pump inhibitors (PPIs) are a widely used class of drugs that function by raising gastric pH. PPIs are an effective short-term indicated therapy for gastroesophageal reflux, peptic ulcers, and *H. pylori* infection, but many chronically afflicted patients take long-term or off-label dosing. Meta-analyses have shown that PPI use increases the risk of developing SIBO and *C. difficile* infection (odds ratios: 1.71 and 1.99; 95% confidence intervals: 1.20–2.43 and 1.73–2.30, respectively) [288,289]. Antibiotics, PPIs, and atypical antipsychotics have each been implicated in reducing alpha microbial diversity [286,290,291]. Second-generation antipsychotic medications, which contribute to weight gain and metabolic syndrome, gradually increase the ratio of Firmicutes to Bacteroidetes in association with BMI and decrease the abundance of *Akkermansia* [292,293]. Efforts are now underway to examine how bacterial taxa each respond to treatment with drugs from other common therapeutic classes [290,294]. Opioids can cause severe constipation and at high doses in mice enable bacterial translocation through disruption of the gut barrier [290,295]. Changes in microbiota have been implicated in the creation of intestinal lesions by nonsteroidal anti-inflammatory drugs, which reduce blood flow to the gut and weaken the hydrophobic mucosal barrier. Lastly, GI symptoms are a common side effect of statins, which affect bile acid metabolism and have been shown to increase the abundance of five bacterial families including Enterobacteriaceae [290].

The interrelationships discussed in this article between diet, environmental factors, gut microbiota, and their physiological outcomes are summarized in Table 1.

## 8. Conclusions and Future Directions for Research

The past decade of research has begun to reveal the overarching roles the gut microbiome plays in human health. Particular species of *Bifidobacterium*, *Akkermansia*, and *Lactobacillus* are beneficial to the human host and are included in many probiotic preparations, but genera such as *Bacteroides* and *Ruminococcus* are implicated in negative health outcomes. Antibiotic use and modern sanitation have contributed to a decrease in the diversity of the human microbiome [287]. Core microbial diversity and the ratio of Firmicutes to Bacteroidetes are general indicators of health and may change with age, though inter-individual variation is large and quality of diet and environmental factors play a dominant role [246,297,298,299]. Future research will need to characterize the changes in bacterial composition accompanying different disease states and the corresponding expression patterns in genes of both microbe and host [296,300]. Increased age is associated with oxidative stress and a pro-inflammatory state, and improvements in microbiota have been shown to extend life span in animal models of aging, though human aging studies are lacking [81,278,301,302]. Prebiotics and dietary fiber increase the relative abundance of beneficial anaerobic bacteria, increase butyrate fermentation, and have favorable metabolic effects. Propionate, on the other hand, is an SCFA used as a food preservative that has recently been linked to insulin resistance when consumed in typical concentrations [303]. Lastly, negative results are being reported for gut microbiota-produced acetate. In rats fed a high-fat diet, increased acetate production was found to promote obesity and metabolic syndrome [304]. In an analogous rat model, colonic infusion with resistant starch plus exogenous acetate delayed the development of obesity and insulin resistance and protected the mucosal barrier [305]. Genera such as *Faecalibacterium* and *Roseburia* were observed to enable the conversion of acetate into butyrate, increasing serum and fecal butyrate levels.

While our knowledge of commensal and pathogenic bacteria has grown considerably, future research will need to further address the role of nonbacterial microbes in the human gut, including viruses, eukaryotes, yeasts, and archaea [256,306,307]. Viruses parasitic to bacteria, known as bacteriophages, have been shown to coexist over time with the bacterial species they prey on. Phage predation can also lead to cascading effects on other species, including blooms in non-targeted bacteria [308]. An abnormal enteric virome has been found in IBD patients, in whom an increase in bacteriophage richness contributes to decreasing bacterial diversity and gut dysbiosis [309]. The most prevalent eukaryote in the human intestine, *Blastocystis*, is a single-celled heterokont protist that colonizes a considerable fraction of individuals in industrialized (0.5–30%) and developing (30–76%) nations [310]. It has been hypothesized that *Blastocystis* can prey on bacterial species in the gut in its ameboid form [306] and can contribute to the pathogenesis of IBS [311]. In a mouse study by Yason et al., infection with a pathogenic subtype of *Blastocystis* (ST7) was observed to decrease intestinal levels of beneficial *Bifidobacterium* and *Lactobacillus* while increasing *E. coli* content, seeming to fulfill Koch’s postulate that infection of a healthy individual leads to disease [312]. In asymptomatic individuals, however, nonpathogenic *Blastocystis* correlates positively with microbial diversity and inversely with BMI, fecal calprotectin levels, Crohn’s disease, and colorectal cancer [313]. As a genus, species of *Blastocystis* have incredibly divergent genomes. The percentage of proteins unique to each subtype ranges from 6% to 20%, and orthologous proteins have a median amino acid sequence identity of only 60% [314].

Diet and nutritional status are important determinants in human health. Efforts to characterize the relationship between diet and health have pivoted from studying the effects of individual nutrients to examining the roles of dietary patterns and specific diets [149,150,151]. The role of diet in shaping gut microbiota, host metabolism, and lipid homeostasis is changing our view of the steps a person can take to make improvements in their systemic health [10,315]. Correlations between microbial diversity across as many as 60 different dietary covariates reveal the importance of a high quality, balanced diet [120], supporting the view that dietary supplementation of individual nutrients does not take the place of a sound diet [316]. Observations that individual foods stimulate the growth of specific bacterial taxa suggest that intestinal bacteria could actually be serving to guide our food preferences, appetite, and feelings of satiety [221,317]. By influencing metabolism and inflammation, diet and nutrition can outweigh genetic and environmental factors in determining health outcomes for chronic Western conditions such as diabetes, obesity, IBS, IBD, colorectal cancer, and depression [1,2,318].

One research question that remains is what constitutes an optimal health-promoting microbiome, and how individuals with different starting microbiota can achieve such microflora. In characterizing gut eubiosis and dysbiosis, the effects of particular microbial species cannot be considered simply in isolation, giving rise to the notion of ecological Koch’s postulates of disease causation [162]. Changes in stool consistency and water content have hampered quantification of absolute microbial loads, and new methods are needed to identify pathological markers [319]. While fecal samples are generally thought to be representative of colonic microbial communities, further research is needed to characterize the different microbial communities that occur along the length of the GI tract [320]. A study of five gut sections taken from pigs found a predominance of *Lactobacillus* in the small intestine and *Prevotella* in the colon, suggesting that rapid utilization of simple carbohydrates drives microbial competition in the upper intestine, while polysaccharide fermentation is left mainly to the colon [321].

Inter-individual variation in gut microbiota could explain the disparity in outcomes often observed with lifestyle interventions and why one-size-fits-all diets are not always effective [83,125,201]. The influence of diet type on the relative abundances of microbial populations can be complex and difficult to reproduce across different clinical studies, in part due to the number of individual species involved in each phylum and genus [142]. Individuals have been shown to have highly personalized microbiome responses to different foods depending on their prior history of dietary diversity [322]. Rapid modifications in gut microbiota are possible when adopting a new dietary strategy, such as following an exclusively plant- or animal-based diet [323]. Microbial markers have even been proposed as an objective means of measuring adherence to a given dietary pattern in order to more accurately correlate resultant health outcomes [150]. Microbes collectively encode 150-fold more genes than the human genome [5]. Enzymes in gut bacteria across the main taxonomic groupings have been shown to metabolize 176 common oral drugs, suggesting that differences in gut microbiota may shape individual responses to drug therapy [324]. Ultimately, determining the full landscape of host-microbiota interactions will enable advances in personalized medicine, precision nutrition [125,325], and the development of next-generation probiotics tailored to the individual [326].

## Figures and Tables

**Table 1:** Summary of diet-microbiota interactions in health and disease.

Healthy Microbiota	Gut Dysbiosis	Other Cause/Consequence
High dietary fiber intake [115]	Western diet; low core diversity [10,83]	High in choline/fat/added sugar [105,117]
Plant foods low in choline [151]	High [TMAO] in blood [134]	Arterial plaque formation [135]
Fruits and vegetables; prebiotic-containing foods [4]	Low fiber intake/low FODMAP carbs [244]	Beer, bread, sugar/artificially-sweetened beverages [120,122]
High α species diversity; butyrate-producing [4,105,120]	Low short-chain fatty acid fermentation [100]	Intestinal inflammation [25,117]
Anti-inflammatory omega-3 [217]	Diet high in omega-6 fatty acids	Pro-inflammatory [149]
Lean body mass, increased lipolysis [84]	Obesity, vagal remodeling, increased energy harvest [85,105]	Increased appetite/lipogenesis [103]
High *Prevotella*/low *Bacteroides*; abundance of *A. muciniphila* [12,14,91]	Abundance of *Ruminococcus* [16,105]	High Firmicutes:Bacteroidetes ratio [85,105]
Glucose and lipid homeostasis [100]	Insulin resistance, bacterial encroachment [76,106,271]	Cardiovascular disease [111,151]
Beneficial bacteria/probiotics: *Bifidobacterium, Lactobacillus* [192,206]	Oxidative stress; facultative anaerobes; *E. coli* [38]	Broad-spectrum antibiotics [22,39,287]; medication dysbiosis [290]
Gut-brain interactions [78]	Mental health issues or visceral pain [72,296]	Leaky gut, plasma endotoxin, psychological stress; emulsifiers [54,272]
Regular intestinal motility [222,259]	Structural or functional bowel disorders [22,50]	Colorectal cancer [3]
Healthy fecal biomarkers [53]	Need butyrate/inulin supplementation [81,104,213]	Potential for fecal transplant [73,76]
Intermittent fasting; adipose beiging [273]	Excess starch/sugar consumption [120]	*Candida* overgrowth; gluten sensitivity [241,256]

## Abbreviations

BMI	body mass index
CB_1_	cannabinoid receptor 1
CVD	cardiovascular disease
DHA	docosahexaenoic acid
DMB	3,3-dimethyl-1-butanol
eCB	endocannabinoid
EPA	eicosapentaenoic acid
FMT	fecal microbial transplantation
FODMAP	fermentable oligo-, di-, mono-saccharides and polyols
FOS	fructo-oligosaccharide
GABA	γ-aminobutyric acid
GI	gastrointestinal
GOS	galacto-oligosaccharide
GPCR	G-protein coupled receptor
GPR109A	niacin receptor 1
GPR41	free fatty acid receptor 3
HR	hazard ratio
HDL	high-density lipoprotein
IBD	inflammatory bowel disease
IBS	irritable bowel syndrome
IBS-C	IBS with predominant constipation
IBS-D	IBS with predominant diarrhea
IBS-M	IBS with alternating bowel habits
KD	ketogenic diet
LDL	low-density lipoprotein
LPS	lipopolysaccharide (endotoxin)
NAS	non-caloric artificial sweetener
NCGS	non-celiac gluten sensitivity
*P*	probability value
PPAR-γ	peroxisome proliferator-activated receptor gamma
PPI	proton pump inhibitor
PUFA	polyunsaturated fatty acid
*R*	Pearson correlation coefficient
RYGB	Roux-en-Y gastric bypass
SCD	specific carbohydrate diet
SCFA	short-chain fatty acid
sdLDL	small dense low-density lipoprotein particle
SGLT1	sodium glucose cotransporter-1
SIBO	small intestinal bacterial overgrowth
THC	tetrahydrocannabinol
TMA	trimethylamine
TMAO	trimethylamine *N*-oxide
TNF	tumor necrosis factor
T2D	type 2 diabetes
